# Disparities in Cervical Cancer Mortality Rates as Determined by the Longitudinal Hyperbolastic Mixed-Effects Type II Model

**DOI:** 10.1371/journal.pone.0107242

**Published:** 2014-09-16

**Authors:** Mohammad A. Tabatabai, Jean-Jacques Kengwoung-Keumo, Wayne M. Eby, Sejong Bae, Juliette T. Guemmegne, Upender Manne, Mona Fouad, Edward E. Partridge, Karan P. Singh

**Affiliations:** 1 School of Graduate Studies and Research, Meharry Medical College, Nashville, Tennessee, United States of America; 2 Department of Mathematics, Cameron University, Lawton, Oklahoma, United States of America; 3 Department of Mathematics, New Jersey City University, Jersey City, New Jersey, United States of America; 4 Division of Preventive Medicine and Comprehensive Cancer Center, University of Alabama at Birmingham, Birmingham, Alabama, United States of America; 5 Department of Economics, University of New Mexico, Albuquerque, New Mexico, United States of America; 6 Department of Pathology and Comprehensive Cancer Center, University of Alabama at Birmingham, Birmingham, Alabama, United States of America; 7 Department of Obstetrics & Gynecology and Comprehensive Cancer Center, University of Alabama at Birmingham, Birmingham, Alabama, United States of America; Texas Tech University, United States of America

## Abstract

**Background:**

The main purpose of this study was to model and analyze the dynamics of cervical cancer mortality rates for African American (Black) and White women residing in 13 states located in the eastern half of the United States of America from 1975 through 2010.

**Methods:**

The cervical cancer mortality rates of the Surveillance, Epidemiology, and End Results (SEER) were used to model and analyze the dynamics of cervical cancer mortality. A longitudinal hyperbolastic mixed-effects type II model was used to model the cervical cancer mortality data and SAS PROC NLMIXED and Mathematica were utilized to perform the computations.

**Results:**

Despite decreasing trends in cervical cancer mortality rates for both races, racial disparities in mortality rates still exist. In all 13 states, Black women had higher mortality rates at all times. The degree of disparities and pace of decline in mortality rates over time differed among these states. Determining the paces of decline over 36 years showed that Tennessee had the most rapid decline in cervical cancer mortality for Black women, and Mississippi had the most rapid decline for White Women. In contrast, slow declines in cervical cancer mortality were noted for Black women in Florida and for White women in Maryland.

**Conclusions:**

In all 13 states, cervical cancer mortality rates for both racial groups have fallen. Disparities in the pace of decline in mortality rates in these states may be due to differences in the rates of screening for cervical cancers. Of note, the gap in cervical cancer mortality rates between Black women and White women is narrowing.

## Introduction

With proper screening and early intervention, cervical cancer, caused by infection with particular types of human papillomavirus (HPV), is a highly treatable disease. Because of the screening process and the long period for cancer development, it is also generally preventable. Mortality rates, which have been steadily decreasing with the advent of improved treatment/screening methods, provide a measure of the success in the treatment and/or screening modalities of cervical cancer. A primary focus in modeling cervical cancer mortality rates is the disparity of these treatment/screening results among different races. In particular, we were interested in analyzing the disparity in cervical cancer mortality between White and African American (Black) women, and we performed a longitudinal study with respect to variation of these outcomes in 13 states. We expected to find differences in the mortality rates between these ethnic groups because of the issues of screening, socioeconomic status, education, access to treatment, general health, obesity, and other confounders. We also expected to find variation in these confounders, at a smaller level, from state to state. For this reason, our longitudinal study was applied as a mixed-effects model.

Hyperbolastic, logistic, and Gompertz models are applicable to biological modeling, including rates of survival, incidence, and mortality. The Gompertz model has been applied to assess differences in mortality rate increases between White and Black populations [1] and for genomic selection for longitudinal data [2]. These models are also applied in longitudinal studies wherein the mixed-effects models are routinely used. Novák et al. [3] applied the logistic growth model to longitudinal growth data for children and adolescents. Aggrey [4] reported a longitudinal study of growth weights using logistic mixed-effects models, and Li and Jiang [5] conducted a similar study to measure tree diameters.

Here, to analyze cervical cancer data, we applied the longitudinal hyperbolastic of type II mixed-effects model (H2), previously utilized by Tabatabai *et al.* [6]. Hyperbolastic models add flexibility of shape and have proven useful in various biomedical applications, including measuring cellular proliferation [7], [8] and wound healing [9]. These models provide a flexible means to present mortality rates as a function of time and covariates. Hyperbolastic models have been presented in a multivariable form, allowing the use of one or more additional explanatory variables [9], [10]. In the present investigation, the H2 model was used to allow assessment of the inherent variability between individual subjects as well as within a subject.

To evaluate the role of race on the mortality rates for cervical cancer, a longitudinal study was performed for thirteen states available data in the eastern half of the USA. Such a longitudinal, multi-level study allows comparison of the relative importance of race in these states. It was expected that incidence and mortality rates would vary across different geographical regions due to differences in prevalence of HPV DNA in women in these states. The mixed-effects model allows for variability in some of the model parameters to account for the regional variation and, in addition, variation within each state over time. A mixed-effects model improves accuracy, and the built-in variability of these model parameters allows for the influence of factors other than race, not explicitly described in the model. These models were applied to data on the mortality rates for women with cervical cancer found in the Surveillance, Epidemiology, and End Result (SEER) program of the National Cancer Institute (NCI) [11].

Even though there is widespread use of screening (Pap smears and HPV DNA testing) to detect precancerous lesions of cervical cancer, racial disparities remain in both incidence and mortality rates, which are higher among Hispanic and Black women relative to White women. In fact, Black women have experienced the largest decreases in mortality rates since 1992 but still have rates more than twice that of White women [12]. With our model, we were able to view this long-term decrease in mortality rates and the corresponding racial disparities and also to measure the pace of decline in cervical cancer mortality as an assessment of disparity.

Since the risk of cervical cancer and racial disparity are associated with socioeconomic status and become relevant in studies correcting for comorbid conditions and other influences [25], we assessed the value of socioeconomic status in cervical cancer risk and its relation to other risk factors. We also report the findings of a study that applied the longitudinal H2 model to estimate the cervical cancer mortality rates for Black and White women in 13 states (Alabama, Florida, Georgia, Illinois, Louisiana, Maryland, Mississippi, New York, North Carolina, Pennsylvania, South Carolina, Tennessee, and Texas), which are located in the eastern half of the United States of America. In addition, for each individual state, an analysis of disparities in cervical cancer mortality rates was performed.

## Methods

We applied the H2 model, which is useful to determine whether there are differences between individual states as well as differences within each state with regard to mortality rates. In general, a nonlinear mixed-effects model has a form
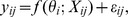



where *y_ij_* is the *jth* response on the *ith* subject, *X_ij_* is the vector of covariates for the *jth* response on the *ith* subject, *ε_ij_* is the normally distributed noise term, α is a vector of fixed parameters, and *b_i_* is the vector of random effects with mean vector 0 and variance-covariance matrix *V*. The vector *θ_i_* is defined as
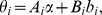



where *A_i_* and *B_i_* are design matrices for α and *b_i_* respectively.

For the analysis of cervical cancer data, we used the H2 model, which has the form
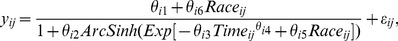
(1)


where the covariate Race is 1 if the person is a White woman and 0 if she is a Black woman. The variable year here is denoted by Time. Time takes the value of 1 corresponds to the year 1975. The mixed- and the fixed-effects vectors, *α* and *b_1_*, for the cervical cancer data are
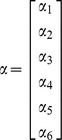



and 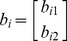



and the design matrices are 
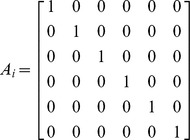



and
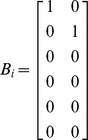



with
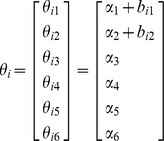



Use of the above information results in the following equation
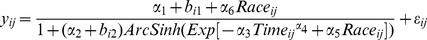
(2)


We assumed that the random effects vector 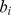 has a bivariate normal distribution with mean vector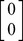 and variance-covariance matrix 
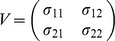
.

When *θ_i5_ = θ_i6_* = 0, the model becomes the H2 model [6]. SAS (Cary, NC) code for analysis with the H2 model is available upon request.

## Results

To eliminate disparities, there is a need (a) to understand the dynamics of mortality for individual states as well as for all states as a whole; (b) to know how fast the mortality rate due to cervical cancer changes with respect to time; (c) to assess, for different groups, whether the gap is widening or narrowing at different points in time; and (d) to identify factors that contribute to the pace of decline. **Tables 1** **and** **2** give the summary statistics on the cervical cancer mortality rates of the 13 states for Blacks and Whites, respectively. As defined by the NCI, the mortality rate is 
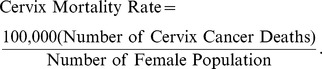



**Table 1:** Summary statistics for Black cervical cancer mortality rates in thirteen U.S. states from 1975 to 2010.

State	Mean	Standard deviation	CV	Minimum	Lower quartile	Median	Upper quartile	Maximum
*A*L	9.30	4.05	43.55	4.10	5.65	8.45	11.85	17.80
FL	8.94	3.51	39.26	4.00	5.50	8.75	12.05	14.90
GA	7.46	3.17	42.49	3.60	4.70	6.80	9.25	15.40
IL	8.74	2.82	32.27	4.60	6.25	8.50	11.10	14.40
LA	8.06	2.61	32.38	3.90	5.80	8.10	9.60	13.70
MD	6.80	3.11	45.74	2.90	4.15	6.30	8.10	14.80
MS	8.72	2.21	25.34	4.70	7.20	8.60	10.30	13.40
NY	7.33	3.04	41.47	3.20	5.05	6.40	8.85	14.90
NC	8.45	4.30	0.89	3.40	4.25	8.15	10.45	19.50
PA	6.78	2.33	34.37	2.70	5.35	6.80	8.15	11.80
SC	8.54	3.23	37.82	4.10	5.65	8.00	10.90	15.70
TN	9.02	3.20	35.48	3.20	6.20	8.85	11.25	17.30
TX	7.67	2.48	32.33	3.70	5.70	7.70	9.15	13.80

Mortality rates were calculated as defined in the text.

**Table 2:** Summary statistics for White cervical cancer mortality rates in 13 U.S. states from 1975 to 2010.

State	Mean	Standard deviation	CV	Minimum	Lower quartile	Median	Upper quartile	Maximum
AL	3.45	1.14	33.04	2.00	2.55	3.00	4.55	6.00
FL	3.02	0.72	23.84	2.10	2.35	3.00	3.40	5.50
GA	3.43	0.74	21.57	2.40	2.90	3.30	3.70	4.60
IL	3.09	0.71	22.98	2.10	2.50	3.00	3.60	5.20
LA	2.86	0.72	25.17	1.60	2.50	2.75	3.25	6.20
MD	2.82	1.13	40.07	1.40	2.05	2.40	3.25	5.20
MS	2.90	0.74	25.52	1.70	2.45	2.80	3.20	4.40
NY	3.00	0.69	23	1.80	2.35	3.05	3.45	4.90
NC	2.91	0.85	29.21	1.60	2.25	2.75	3.60	4.70
PA	2.92	0.70	23.97	1.80	2.40	2.95	3.30	5.10
SC	3.12	1.00	32.05	1.30	2.40	2.95	4.00	6.00
TN	3.57	1.04	29.13	1.90	2.90	3.30	4.15	5.10
TX	3.45	0.61	17.68	2.50	3.00	3.50	3.65	5.50

Mortality rates were calculated as defined in the text.

The data used for the construction of **Tables 1** **and** **2** are the mortality rates for each state over 36 years, starting with 1975 and ending with 2010.

**Tables 3** **and** **4** show cervical cancer summary statistics for 1975-2010 for Blacks and Whites. In constructing these tables, we took into account the cervical cancer mortality rates for all 13 states. For each year, we give summary statistics for the cervical cancer mortality rates of these states.

**Table 3:** Summary statistics Black cervical cancer mortality by year in thirteen U.S. states.

Year	Mean	Standard deviation	CV	Minimum	Lower quartile	Median	Upper quartile	Maximum
1975	13.95	2.61	18.71	9.70	12.40	13.80	14.90	17.70
1976	12.95	2.73	21.09	9.40	11.20	12.10	14.70	19.50
1977	12.88	2.22	17.24	9.60	11.60	13.00	14.40	17.80
1978	12.90	2.35	18.22	8.50	11.80	12.40	14.60	17.70
1979	10.75	2.44	22.7	6.90	9.00	10.20	13.00	14.40
1980	11.46	2.10	18.33	8.70	9.50	11.70	13.00	14.40
1981	10.38	2.07	19.95	7.70	8.90	10.00	11.60	14.40
1982	10.46	2.06	19.7	7.20	8.30	10.70	11.80	13.50
1983	10.56	1.92	18.19	7.40	8.40	11.30	11.50	13.40
1984	10.44	1.69	16.19	7.70	9.40	10.30	11.60	13.00
1985	9.75	1.72	17.65	7.10	8.20	9.70	11.30	12.00
1986	10.18	1.90	18.67	7.10	8.80	10.10	11.10	13.80
1987	8.72	1.61	18.47	6.20	7.80	8.80	9.50	11.80
1988	8.52	2.14	25.12	5.00	7.50	8.00	9.90	11.80
1989	9.15	2.17	23.72	6.10	7.90	8.60	10.30	13.20
1990	8.31	1.53	18.42	5.00	7.50	8.50	8.70	10.90
1991	8.19	1.44	17.59	6.30	7.60	7.90	8.70	11.40
1992	8.93	1.99	22.29	5.70	7.10	9.10	10.30	12.00
1993	8.54	1.21	14.17	7.00	7.50	8.30	9.30	10.70
1994	7.38	1.10	14.91	5.50	6.80	7.60	8.10	8.80
1995	7.74	1.52	19.64	5.50	6.60	7.90	8.40	11.20
1996	6.75	1.33	19.71	5.00	5.70	6.20	8.00	8.90
1997	7.05	0.93	13.2	6.00	6.40	6.90	7.80	8.70
1998	6.23	0.98	15.74	4.90	5.50	6.20	6.90	8.00
1999	5.80	1.16	20	3.80	5.30	6.00	6.40	8.20
2000	6.00	1.61	26.84	3.30	5.20	5.80	7.60	8.40
2001	5.18	1.40	27.03	3.20	4.10	4.70	5.70	8.00
2002	5.51	0.99	17.97	3.90	4.70	5.60	6.10	7.40
2003	5.15	1.32	25.64	3.50	3.90	5.20	5.70	8.20
2004	5.25	1.06	20.2	3.80	4.40	5.50	5.70	7.60
2005	4.95	1.02	20.61	3.10	4.60	4.80	5.60	6.90
2006	4.85	1.34	27.63	3.40	4.30	4.50	5.20	8.60
2007	4.68	1.26	26.93	2.70	4.00	4.50	5.00	8.00
2008	4.72	1.22	25.85	3.20	3.70	4.70	5.60	7.00
2009	4.55	0.75	16.49	3.60	4.10	4.40	4.70	6.40
2010	4.20	0.99	23.58	2.90	3.60	3.90	4.90	6.20

Mortality rates were calculated as defined in the text.

**Table 4:** Summary statistics White cervical cancer mortality by year in thirteen U.S. states.

Year	Mean	Standard deviation	CV	Minimum	Lower quartile	Median	Upper quartile	Maximum
1975	4.88	0.75	15.37	3.90	4.40	4.70	5.10	6.20
1976	4.78	0.58	12.14	3.90	4.40	4.70	5.20	5.60
1977	4.37	0.73	16.71	3.10	4.10	4.30	4.60	5.90
1978	4.35	0.54	12.42	3.60	4.10	4.30	4.30	5.30
1979	3.97	0.69	17.39	3.00	3.60	3.90	4.20	5.30
1980	3.88	0.74	19.08	2.40	3.50	3.80	4.20	5.00
1981	3.79	0.40	10.56	3.20	3.50	3.80	4.00	4.50
1982	3.58	0.72	20.12	2.30	3.40	3.50	3.90	4.70
1983	3.52	0.52	14.78	2.90	3.10	3.40	4.00	4.60
1984	3.65	0.78	21.37	2.10	3.30	3.60	3.70	5.40
1985	3.41	0.47	13.79	2.40	3.30	3.40	3.60	4.20
1986	3.19	0.58	18.19	2.00	3.10	3.20	3.50	4.00
1987	3.36	0.64	19.05	2.40	3.10	3.30	3.60	4.90
1988	3.08	0.52	16.89	2.20	2.80	3.10	3.30	4.20
1989	3.11	0.47	15.12	2.60	2.90	3.00	3.10	4.50
1990	3.08	0.45	14.62	2.30	2.70	3.10	3.50	3.70
1991	2.96	0.34	11.49	2.20	2.90	3.00	3.10	3.50
1992	3.06	0.42	13.73	2.40	2.80	3.00	3.30	3.80
1993	3.03	0.41	13.54	2.40	2.80	3.00	3.30	3.70
1994	3.15	0.45	14.29	2.70	2.90	3.00	3.30	4.20
1995	2.83	0.41	14.49	2.10	2.60	2.80	3.10	3.50
1996	2.85	0.33	11.58	2.40	2.70	2.90	3.10	3.50
1997	2.97	0.45	15.16	2.50	2.70	2.80	3.20	4.10
1998	2.78	0.48	17.27	1.90	2.60	2.90	3.10	3.70
1999	2.72	0.33	12.14	2.00	2.60	2.70	2.90	3.30
2000	2.54	0.54	21.26	1.30	2.40	2.50	2.90	3.50
2001	2.49	0.40	16.07	1.80	2.30	2.40	2.70	3.10
2002	2.45	0.45	18.37	1.70	2.20	2.30	2.70	3.40
2003	2.25	0.56	24.89	1.40	1.90	2.20	2.40	3.50
2004	2.26	0.54	23.9	1.40	1.90	2.20	2.40	3.30
2005	2.35	0.41	17.45	1.50	2.20	2.40	2.70	2.90
2006	2.31	0.41	17.75	1.80	2.00	2.30	2.50	3.10
2007	2.35	0.36	15.32	1.70	2.10	2.40	2.50	2.90
2008	2.38	0.39	16.39	1.80	2.10	2.40	2.70	3.00
2009	2.25	0.51	22.67	1.70	2.10	2.10	2.40	3.70
2010	2.24	0.40	17.86	1.60	2.00	2.30	2.50	2.90

Mortality rates were calculated as defined in the text.

Use of formula (2) and SAS PROC NLMIXED enabled us to perform the H2 model. The output is summarized in **Table 5**. The variance covariance matrix for the mixed-effects model is given by
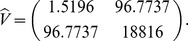



**Table 5:** Parameter estimates for the generalized H2 model (SAS output).

Parameter estimates									
Parameter	Estimate	SE	DF	t-value	P-value	Alpha	Lower	Upper	Gradient
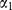	13.1361	0.3877	24	33.89	<.0001	0.05	12.3360	13.9361	0.000811
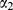	553.72	103.74	24	5.34	<.0001	0.05	339.62	767.82	0.005632
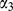	12.6426	0.4053	24	31.20	<.0001	0.05	11.8062	13.4790	0.001518
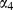	−0.2230	0.01105	24	−20.18	<.0001	0.05	−0.2457	−0.2002	0.019119
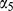	−0.5316	0.1402	24	−3.79	0.0009	0.05	−0.8210	−0.2421	−0.00066
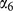	−8.7847	0.5171	24	−16.99	<.0001	0.05	−9.8520	−7.7173	0.000311
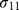	1.5196	0.4481	24	3.39	0.0024	0.05	0.5947	2.4445	−0.00011
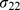	18816	1.6293	24	11548.6	<.0001	0.05	18813	18820	−0.00014
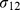	96.7737	31.6337	24	3.06	0.0054	0.05	31.4849	162.06	−0.00004
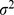	1.0442	0.04962	24	21.05	<.0001	0.05	0.9418	1.1467	−0.00014

**Table 5** shows that all parameters in the model are highly significant.

**Figure 1:**
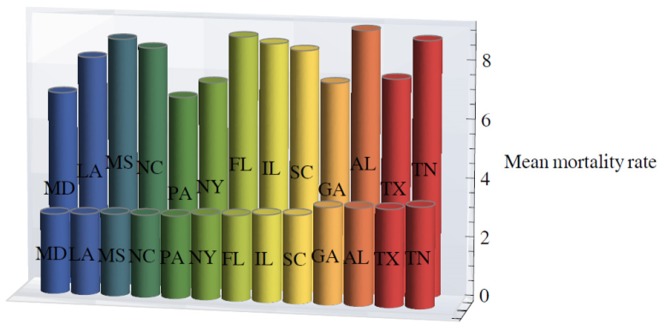
Mean mortality rates for White (front row) and Black (back row) women for the thirteen U.S. states from 1975 through 2010. Mortality rates were calculated as defined in the text.

**Figure 2:**
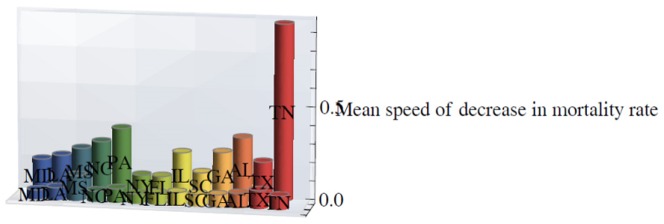
Mean pace of decrease in mortality rates for White (front row) and Black (back row) women for the thirteen U.S. states from 1975 through 2010.

**Figure 3:**
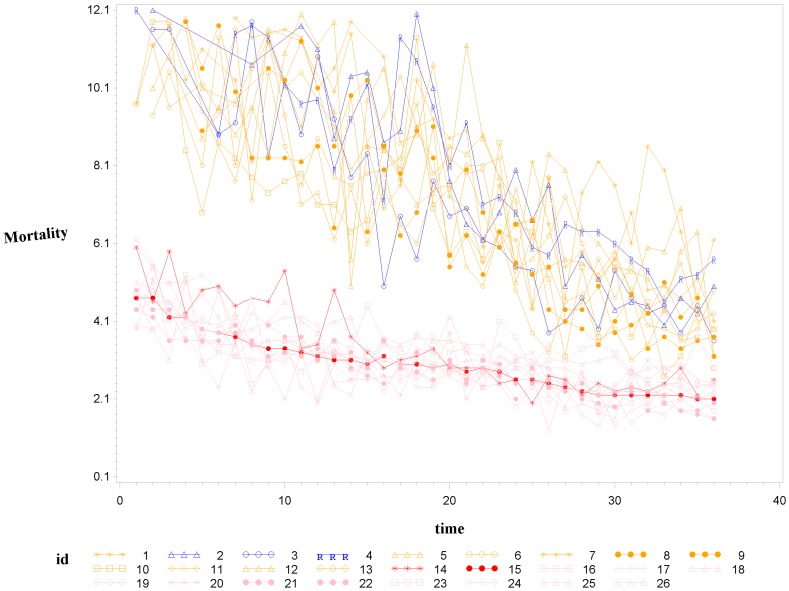
Actual data for cervical cancer mortality rates for Black and White women.

**Figure 4:**
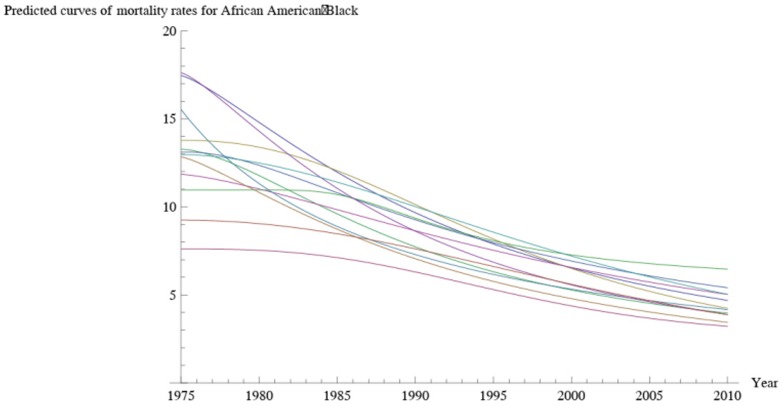
Predicted values for cervical cancer mortality rates for Black women.

**Figure 5:**
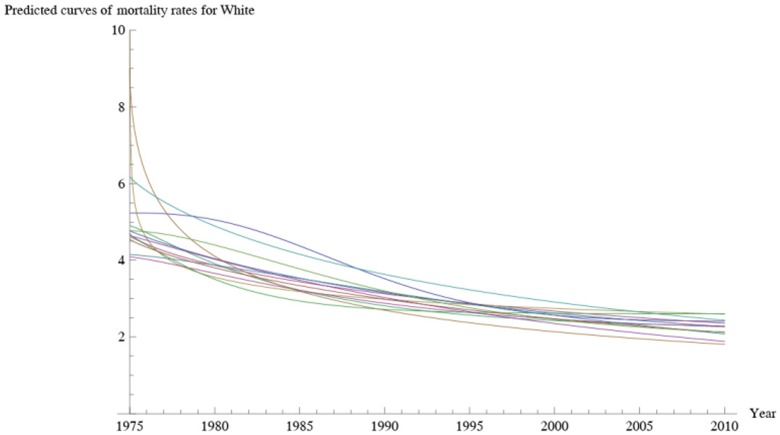
Predicted data values for cervical cancer mortality rates for White women.

**Figure 6:**
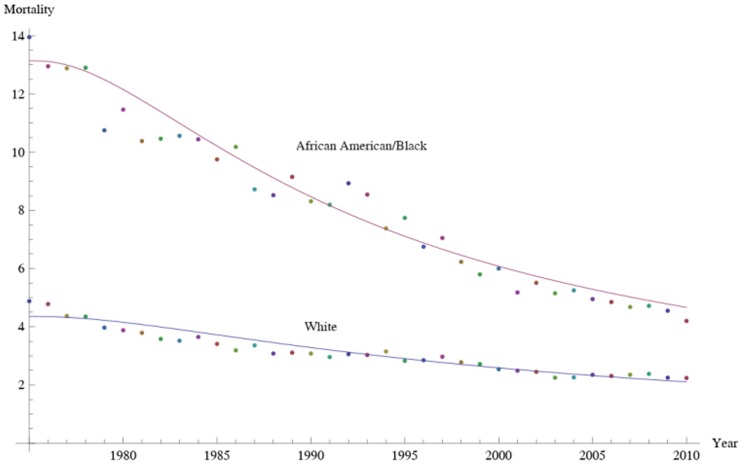
Cervical cancer mortality rates for Black and White women as determined by the H2 model.

**Figure 1** shows 3D bar graphs for cervical cancer mortality of all 13 states during the entire period of 1975–2010, and **Figure 2** shows 3D bar graphs for the pace of decline in cervical mortality rates for all 13 states. **Figure 3** shows a graph of the actual lines for mortality rates in all 13 states. The top portion of the graph represents the cervical cancer mortality for Black women, and the lower portion shows the mortality for White women. **Figure 4** shows the curves for Black women fitted for all 13 states by use of the H2 model and SAS PROC NLMIXED; **Figure 5** shows similar curves for White women. **Figure 6** shows the fitted curves for the average of all 13 states for Black women and White women.

### State-level analyses of cervical cancer mortality rates

#### Alabama

**Figure 7:**
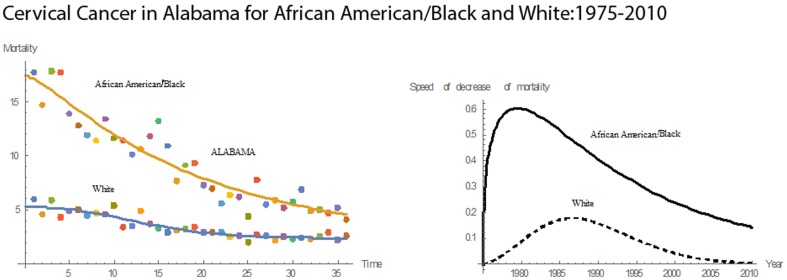
Mean mortality rates, pace of decrease, and 3D histograms for Black and White women in Alabama, 1975–2010.

For Alabama, the cervical cancer mortality rates for both Whites and Blacks declined during the period of 1975–2010 **(** **Figure 7** **)**. Alabama ranked highest among the 13 states in mean cervical cancer mortality for Blacks and had the third highest mortality for Whites. Also, Alabama had the third highest rate in mean pace of decline in mortality for both races. Over the 36 years, the mean mortality rate was 9.30 for Blacks and 3.45 for Whites. In this time, the cervical cancer mortality rates decreased for both races. **Figure 7** illustrates the mortality trends as well as the pace of decline in mortality. The mortality rate for Black women in Alabama ranged from a high of 17.80 to a low of 4.10. The estimated median cervical cancer mortality rate for Blacks and Whites in Alabama was 8.45 and 3.00, respectively. The pace of the decline in mortality rates for Blacks rose until reaching its top pace of 0.60 in 1979. After that year, the pace fell. For White Alabamans, the pace of decline in cervical cancer mortality rose until 1986, at which time the maximum pace of 0.176 was reached; thereafter, the pace of decline in mortality fell. In 1975, the mortality rate for Blacks was approximately three times that for Whites. The gap narrowed steadily after 1975, and in 2010 the mortality rate for Blacks became twice that for Whites. The pace of decline in the mortality curve for Blacks stayed above that for Whites throughout the period. Thus, although the gap in mortality rates in Alabama has narrowed, a disparity remains.

#### Florida

**Figure 8:**
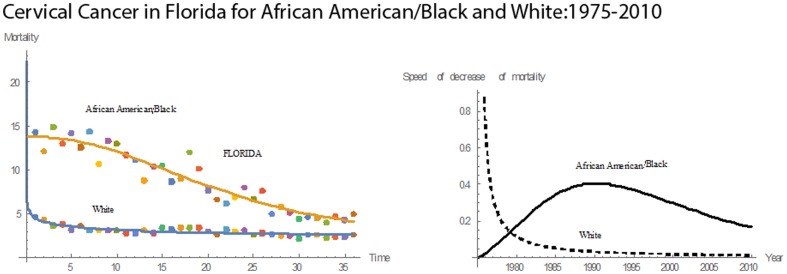
Mean mortality rates, pace of decrease, and 3D histograms for Black and White women in Florida, 1975–2010.

In Florida from 1975 to 2010, despite a decline in cervical cancer mortality rates for Whites and Blacks, Blacks continued to suffer the greatest burden **(** **Figure 8** **)**. Compared to the other states, Florida had the third highest rate in mean cervical cancer mortality for Blacks and seventh highest for Whites. Also, Florida had the thirteenth highest rate in mean pace of decline in cervical cancer mortality for Black women and the tenth highest for White women. In 36 years, Florida had mean mortality rates of 8.94 for Blacks and 3.43 for Whites. For both Whites and Blacks, the state had the third highest mean mortality rate among all 13 states. The mortality rate for Blacks ranged from a low of 4.00 to a high of 14.90. For Whites, it ranged from 2.10 to 5.50. For White women, the pace of decline in mortality decreased steadily after 1975 **(** **Figure 2** **)**. For Blacks, the pace of decline increased until 1992, at which time it reached its maximum value; from 1992 until 2010, the pace of decline had a downward trend. During 1975–1983, pace of decline in Whites was higher than that for Blacks. In 1983, both races had an equal pace of decline of 0.082. In 1975, the cervical cancer mortality rate for Blacks was three times that for Whites, but in 2010 the rate for Blacks was only 2.4 times that for Whites.

#### Georgia

**Figure 9:**
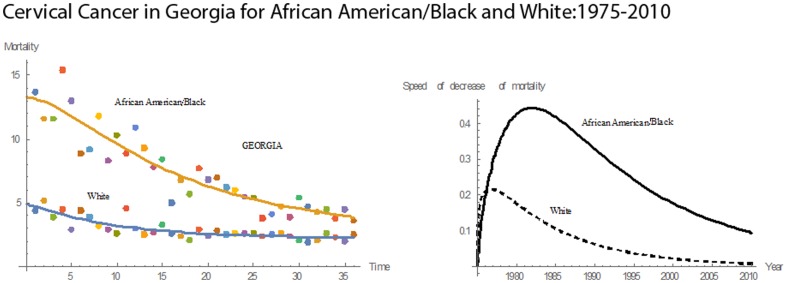
Mean mortality rates, pace of decrease, and 3D histograms for Black and White women in Georgia, 1975–2010.

For 1975–2010, Georgia had a steady decrease in mortality rates for both Blacks and Whites **(** **Figure 9** **)**. The mean mortality rate for Blacks was 7.46 and 3.43 for Whites. Georgia had the third highest cervical cancer mortality rate among the 13 states studied. Over 36 years, the median cervical cancer mortality rate for Blacks was 6.80; for Whites, the rate was 3.30. In 1975, the mortality rate for Blacks was 3 times that for Whites, but, in 2010, it was reduced to 1.7 times that for Whites. From 1975 through 1979, the pace of decline in cervical cancer mortality for Whites was greater than that for Blacks **(** **Figure 3** **)**. In 1979, Blacks and Whites had the same pace of decline, 0.1391. After 1979, the decline in mortality for Blacks was higher than that for Whites. From 1975 through 1989, the pace of decline in mortality for Blacks increased. In 1989, it reached a maximum and declined afterwards. Of the states studied, Georgia had the tenth highest rate in cervical cancer mortality for Blacks and the fourth highest mortality for Whites. Also, Georgia had the fifth highest mean pace of decline in the mortality rate for Blacks and eighth highest for Whites.

#### Illinois

**Figure 10:**
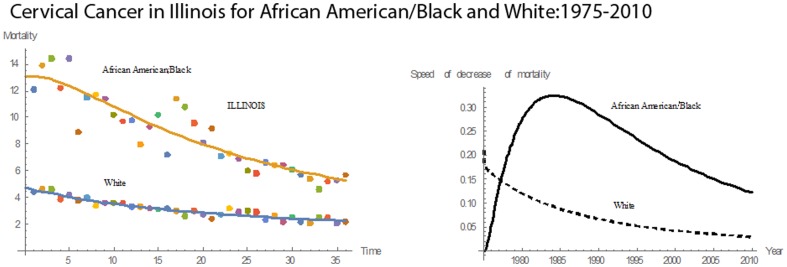
Mean mortality rates, pace of decrease, and 3D histograms for Black and White women in Illinois, 1975–2010.

In Illinois, from 1975 through 2010, the cervical cancer mortality rates for both races had downward slopes **(** **Figure 10** **)**. In 1975, the rate for Blacks was 2.75 times that for Whites, but in 2010 it was reduced to 2.60. In 1975, Blacks had a mortality rate of 12.1; in 2010, it was 5.7. For Whites in 1975, the cervical cancer mortality rate was 4.4; in 2010, it was 2.2. In 36 years, the cervical cancer mortality rate for Blacks dropped by 53%; for Whites, the decrease was 50%. In 1975, the pace of decline in the mortality rate for Blacks began an upward trend that continued until 1981. Thereafter, the pace of decline fell. For Whites, there was an early rise, but after 1976 it had a downward trend. For the entire time, the pace of decline for Blacks was higher than that for Whites. The mean mortality rate was 8.74, and the median was 8.50. Compared to the other states, Illinois had the fourth highest rate in cervical cancer mortality for Blacks and the sixth highest mortality for Whites. Also, Illinois had the seventh highest rate in mean pace of decline in mortality for Blacks and the sixth highest for Whites.

#### Louisiana

**Figure 11:**
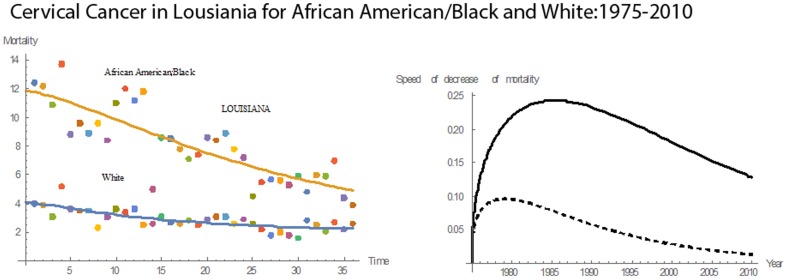
Mean mortality rates, pace of decrease, and 3D histograms for Black and White women in Louisiana, 1975–2010.

Cervical cancer mortality trends for both Blacks and Whites in Louisiana followed a downward slope throughout the 36 years, but Blacks consistently had a higher rate than Whites **(** **Figure 11** **)**. In 1975, the rate for Blacks was 3.1 times that for Whites, but in 2010 it was only 1.5 times. From 1975 to 1977, Whites had higher pace of decline in mortality relative to Blacks. After 1977, the trend reversed, and Blacks had higher pace of mortality. This trend continued until 2010. In 1977, the pace of decline for both Whites and Blacks was 0.1463. The pace of decline for Blacks rose until reaching a maximum in 1983. After 1983, the mortality continued to fall. The decrease in mortality rate in 1975–2010 for Blacks was 69%; for Whites it was 35%. The mean mortality rate was 8.06, and the median was 8.10. Relative to the other states, Louisiana had the eighth highest rate for Blacks and twelfth highest for Whites. Also, Louisiana had the ninth highest rate in the mean pace of decline in cervical cancer mortality for Blacks and seventh highest for Whites.

#### Maryland

**Figure 12:**
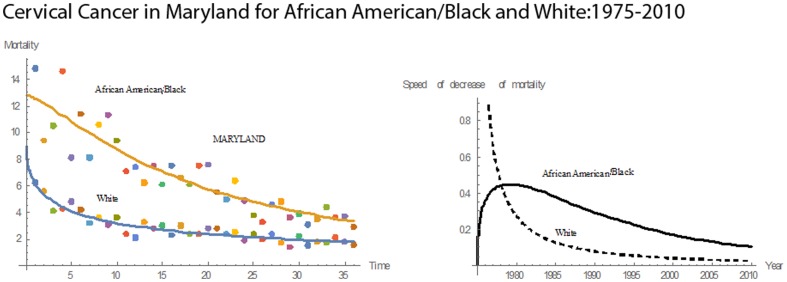
Mean mortality rates, pace of decrease, and 3D histograms for Black and White women in Maryland, 1975–2010.

During 1975–2010, Maryland had decreasing cervical cancer mortality rates for both Blacks and Whites **(** **Figure 12** **)**. In 1975, the rate for Blacks was 14.8; in 2010, it was 2.9, an 80% decrease. In 1975, Whites had a mortality rate of 6.2; in 2010, it was 1.6, a 74% reduction. In 1975, the pace of decline in rates for both Blacks and Whites began to rise. For Blacks, this upward trend continued until 1984, at which time the pace of decline reached its maximum of 0.2427. For Whites, the rise continued until it its maximum of 0.0961 in 1978. After 1978, there was a downward trend. Maryland had a mean cervical cancer mortality rate of 6.80 and a median cervical cancer mortality rate of 6.30. Relative to the other states, Maryland had the twelfth highest rate in mean cervical cancer mortality for Blacks and the thirteenth highest mean mortality for Whites. Also, Maryland had the tenth highest rate in mean pace of decline in cervical cancer mortality for Blacks and the thirteenth highest for Whites.

#### Mississippi

**Figure 13:**
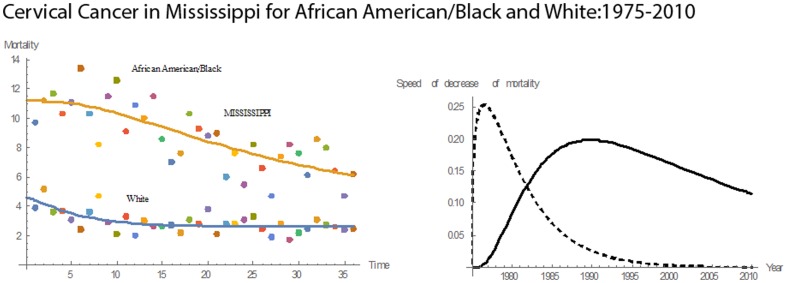
Mean mortality rates, pace of decrease, and 3D histograms for Black and White women in Mississippi, 1975–2010.

For Mississippi, the cervical cancer mortality rates for both races had decreasing slopes during 1975–2010 **(** **Figure 13** **)**. In 1975, the mortality rates for Blacks and Whites were 9.7 and 3.9, respectively. In 2010, these numbers were 6.2 for Blacks and 2.5 for Whites. These represent a 36% reduction for Whites as well as for Blacks. In 1975, the mortality rate for Blacks was 2.5 times that for Whites. During 1975–1978, the pace of decline in the mortality rate for Whites was higher than that for Blacks. In 1978, both Blacks and Whites had the same pace of decline, 0.4419. During 1978–2010, the pace of decline for Blacks was higher than that for Whites. The maximum pace of decline in cervical cancer mortality for Blacks was 0.4513. Mississippi had a mean mortality rate of 8.72 and a median mortality rate of 8.60. This median mortality rate was the third highest for Blacks among the thirteen states. Mississippi had the fifth highest rate in mean cervical cancer mortality rate for Blacks and the eleventh highest for Whites. Also, Mississippi had the sixth highest rate in pace of decline in cervical cancer mortality rate for Blacks and the highest decline for Whites.

#### New York

**Figure 14:**
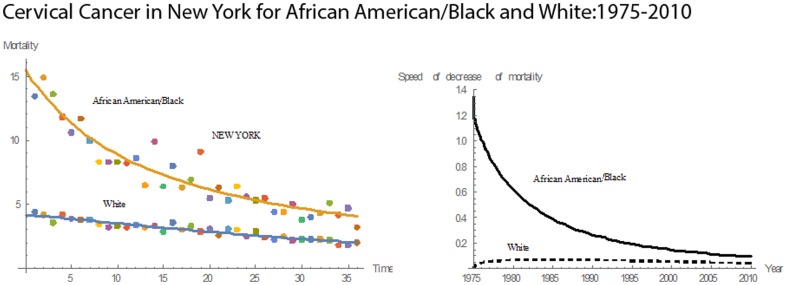
Mean mortality rates, pace of decrease, and 3D histograms for Black and White women in New York, 1975–2010.

For 1975–2010, cervical cancer mortality rates for both Blacks and Whites in New York had downward trends **(** **Figure 14** **)**. In 1975, the rate for was 13.4 for Blacks and for 4.4 for Whites. In 2010, the rates were 3.2 and 2.0, respectively. In 1975, the mortality rate of Blacks was 3 times greater than that of Whites; in 2010, it was 1.6 times greater. From 1975 to 1984, the pace of decline in mortality for Whites was higher than that for Blacks. After 1984, the pace for Blacks was higher than that for Whites until 2010. In 1976, the pace of decline in mortality for Whites reached a maximum of 0.2490; for Blacks, the maximum pace of decline, 0.3040, occurred in 1988. The maximum mean mortality rate was 14.90, and the minimum was 3.20. The mean mortality rate for New York was 7.33, and the median mortality rate was 6.40. New York had the eleventh highest mean mortality rate for Blacks and the eleventh highest for Whites. Also, New York had the twelfth highest rate in mean pace of decline in mortality rate for Blacks and Whites.

#### North Carolina

**Figure 15:**
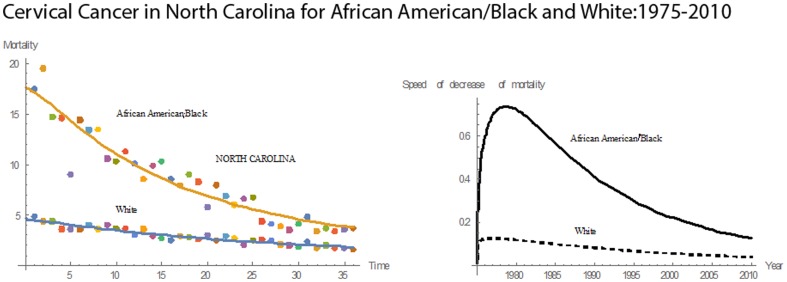
Mean mortality rates, pace of decrease, and 3D histograms for Black and White women in North Carolina, 1975–2010.

North Carolina had a mean mortality rate of 8.45 and a median mortality rate of 8.15. Since 1975, the mortality rates for both races decreased **(** **Figure 15** **)**. The mortality rate for Blacks was higher than that for Whites throughout the 36 years. In 1975, North Carolina had a mortality rate of 17.5 for Blacks and 4.9 for Whites. In 2010, the mortality rates for Blacks and Whites were 3.7 and 1.6, respectively. Between 1975 and 2010, the cervical cancer mortality rate for Blacks decreased by 79% and that for Whites by 67%. In 1975, the cervical cancer mortality rate for Blacks was 3.57 times that for Whites; in 2010, the value was reduced to 2.31. Throughout 1975–2010, the pace of decline in mortality rates for Blacks remained above that for Whites. Relative to the other states, North Carolina had the seventh highest rate in cervical cancer mortality rate for Blacks and tenth highest for Whites. Also, North Carolina had the fourth highest rate in mean pace of decline in mortality for Blacks and eleventh highest for Whites.

#### Pennsylvania

**Figure 16:**
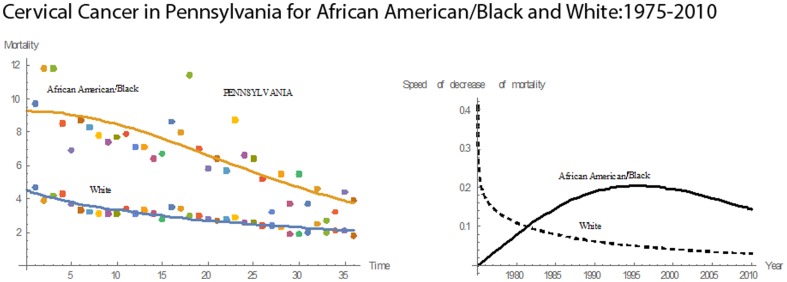
Mean mortality rates, pace of decrease, and 3D histograms for Black and White women in Pennsylvania, 1975–2010.

Throughout the 36 years, Pennsylvania had decreasing cervical cancer mortality rates for both Blacks and Whites **(** **Figure 16** **)**. The mean rates were 6.78 for Blacks and 2.92 for Whites. The corresponding median values were 8.15 for Blacks and 2.95 for Whites. In 1975, the mean mortality rate for Blacks was 9.7; in 2010, it was 3.9. In 1975, for Whites, the mean rate was 4.7; in 2010, it was 1.8. In 2010, the mean mortality rate for Blacks was 2.1 times that for Whites; in 2010, it was 2.2. During 1975–2010, Blacks had a higher pace of decline in cervical cancer mortality. The highest pace for Blacks was 0.736 in 1978. For Whites the maximum pace of decline was 0.126 in 1976. Relative to the other states, Pennsylvania had the thirteenth highest rate in cervical cancer mortality for Blacks and the ninth highest for Whites. Also, Pennsylvania had the second highest rate in mean pace of decline in cervical cancer mortality rate for Blacks and fourth highest for Whites.

#### South Carolina

**Figure 17:**
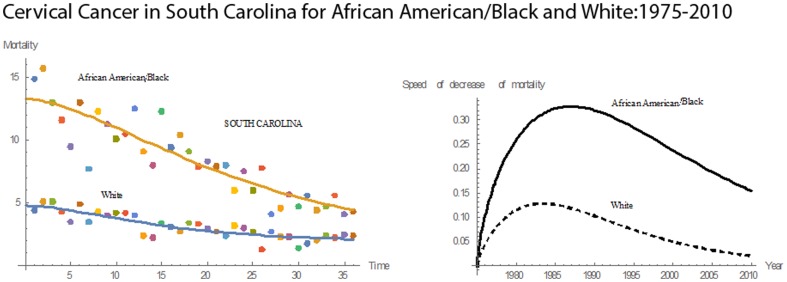
Mean mortality rates, pace of decrease, and 3D histograms for Black and White women in South Carolina, 1975–2010.

For South Carolina, the mean cervical cancer mortality rates for Blacks and Whites over 36 years were 8.54 and 3.12, respectively, and the corresponding median values were 8.00 and 2.95, respectively. Both Blacks and Whites had declining mortality trends **(** **Figure 17** **)**. In 1975, the mortality rate was 14.9 for Blacks and for 4.4 for Whites. In 2010, the rates were 4.3 for Blacks and 2.4 for Whites. During the 36 years, Blacks had a 71% reduction in their mortality rates, and Whites a 45% reduction. In 1975, the mortality rate for Blacks was 3.47 times that for Whites, but by 2010 this number was reduced to 1.79. In 1981, both Blacks and Whites had the same pace of decline, 0.0992. Prior to 1981, the pace of decline in cervical cancer mortality rate was higher for Whites than Blacks, but, after 1981, Blacks had a higher pace of decline. In 1994, the pace of decline was higher for Blacks. Relative to the other states, South Carolina had the sixth highest rate in cervical cancer mortality for Blacks and the fifth highest for Whites. Also, South Carolina had the eleventh highest rate in mean pace of decline in cervical cancer mortality for Blacks and the ninth highest for Whites.

#### Tennessee

**Figure 18:**
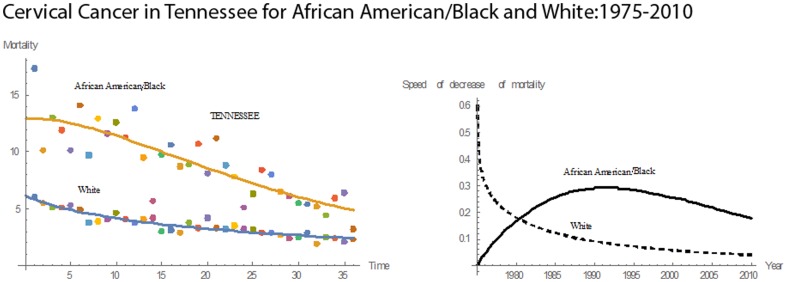
Mean mortality rates, pace of decrease, and 3D histograms for Black and White women in Tennessee, 1975–2010.

Over 36 years, Tennessee had the second highest cervical cancer mean mortality rate of 9.02 for Blacks and the highest rate (3.57) for Whites. Both races had declining mortality rates over this period **(** **Figure 18** **)**. The median mortality rate for Blacks in Tennessee was 8.85, the highest among the 13 states. In 1975, Blacks had a mean mortality of 17.3; the corresponding number for the Whites was 6.00. By 2010, the mortality rates for Blacks and Whites had been reduced to 3.2 and 2.3, respectively. During the 36 years, the reductions in the mortality rates were 82% for Blacks and 62% for Whites. In 1975, Blacks had a mortality rate 2.88 times that for Whites. In 2010, this value was reduced to 1.60. During 1975–2010, the pace of decline in mortality for Blacks was higher than that for Whites. Both Blacks and Whites reached their highest pace of decline in 1982. For Blacks, the magnitude of pace was 1.6995; for Whites, it was 0.1277. Relative to the other states, Tennessee had the highest rate in mean pace of decline in cervical cancer mortality for Blacks and the fifth highest for Whites.

#### Texas

**Figure 19:**
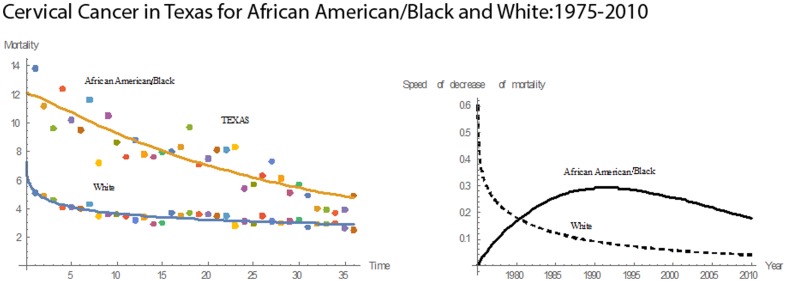
Mean mortality rates, pace of decrease, and 3D histograms for Black and White women in Texas, 1975–2010.

For Texas, both Blacks and Whites had declining rates of cervical cancer mortality for 1975–2010 **(** **Figure 19** **)**. The mean rate for Blacks was 7.67, and the median rate was 7.70. For Whites, the mean mortality rate was 3.45, and the median was 3.50. Texas had the highest median mortality rate for Blacks and the second highest mean mortality for Whites. In 1975, the rate for Blacks was 13.8 and 5.1 for Whites. In 2010, the rates for Blacks and Whites were reduced to 4.9 and 2.5, respectively. In 1975, the mortality rate for Blacks was 2.71 times that for Whites; in 2010, this value was reduced to 1.96. From 1975 to 2010, mortality rates for Blacks and Whites decreased by 64% and 5%, respectively. From 1975 to 1980, the pace of decline in mortality for Whites was higher than that for Blacks. In 1980, both had the same pace of decline of 0.1744. After 1980, the pace of decline for Blacks remained higher. The maximum pace of decline for Blacks was 0.2920, which occurred in 1990. Relative to the other states, Texas had the ninth highest rate in mean cervical cancer mortality for Blacks and the second highest for Whites. Also, the mean pace of decline in mortality was the eighth highest for Blacks and second highest for Whites.

### Combined mortality for 13 states

**Figure 20:**
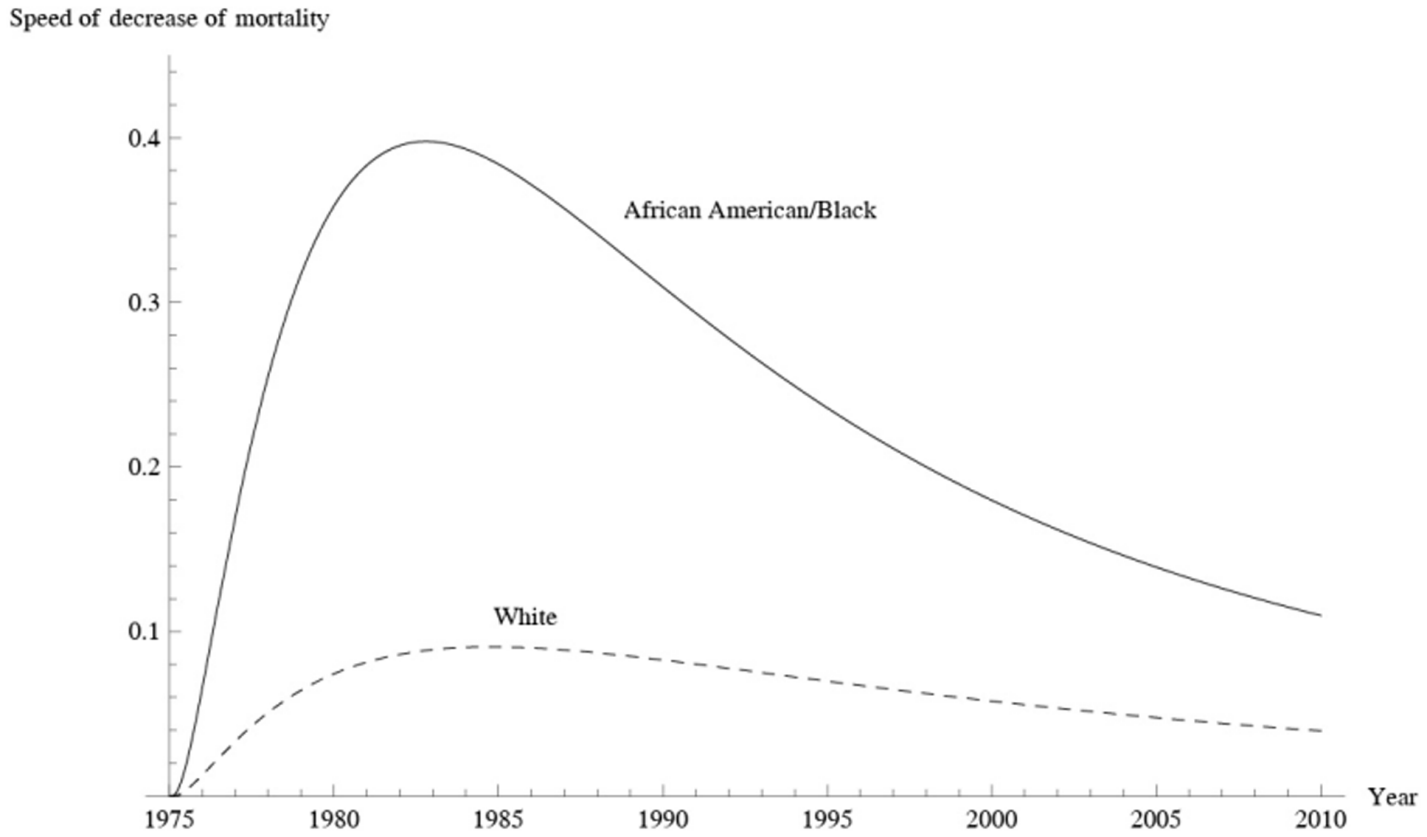
Corresponding pace of decrease in mortality for Black and White women as determined by the generalized H2 model.

For all states, in 1975 the cervical cancer mortality rate was 13.95 for Black women (Table 3) and 4.88 for White women (Table 4). In 2010, the rates for Black and White women were reduced to 4.20 and 2.24, respectively **(** **Figure 20** **)**. The decrease for was 70% for Black women and 54% for White women. In 1975, the mortality rate for Black women was 2.86 times that for Whites, but in 2010 this value was reduced to 1.88. Throughout the 36 years, the cervical cancer mortality rate and the pace of decline in mortality rate for Black women remained higher than those for Whites. For Black women, the pace of decline increased until reaching a maximum of 0.40 in 1983. Afterwards, the pace decreased. For White women, the pace of decline increased until 1984, after which it decreased.

## Discussion

These data reveal a racial disparity in survival rates for cervical cancer. These differences can be attributed to various factors, including biology, access to screening and treatment, stage of the disease at the time of diagnosis, late initiation of treatment, and exposure to risk factors (e.g., HPV infection). This disease is preventable and treatable, assuming appropriate screening and treatment of precancerous lesions. Considerable effort has been made to identify factors underlying the disparity. The efforts to improve outcomes for Black women with respect to cervical cancer have been partly successful in that survival rates have improved; however, a disparity still exists.

Virtually all cases of cervical cancer are caused by HPV infection, which can induce tumorigenesis. Almost all cervical cancer cases are due to infection from the HPV alpha genus [18], which includes the high-risk types, particularly HPV 16 and 18. Although HPV infection is common in the general population, most infections do not lead to cervical cancer, because HPV may require one or more “cofactors” to produce invasive cancer. A high viral load and persistent infection [19], genetics [22], immune response [18], smoking, long term oral contraceptive use and multiple sexual partners [20], a large number of pregnancies [24], and co-infection with sexually transmitted diseases, particularly *Chlamydia trachomatis* and HIV [21] are among the most relevant cofactors.

Approximately 7.5 million US women exhibit HPV infection, an overall rate of 26.8%, with the highest rate of 44.8% found among women 20 to 24 years old. Although a lower percentage of these infections are the high-risk types [13]. Among those cases that become cancerous, the long period between the initial infection and tumor development provides ample time for screening and treatment. The success of the screening programs relate to their capacity to identify lesions in precancerous stages, or in the earliest stages of tumor development, and to the fact that treatments are highly effective and can often prevent development of cancers. Currently, with appropriate treatment, the 5-year survival rate approaches 100% [14]. The survival rates, however, decrease after the cervical carcinomas reach an advanced stage. Although, the survival rates vary among the published studies, they all follow the same trend [15]. Specifically, the survival rates are about 90% for women with stage I, less than 50% for stage II, and about 10% for stage III.

Pap smear testing, a screening process, is useful because it identifies and allows treatment of precancerous lesions or cancers in the earliest stages. It takes considerable time for the precancerous stages, referred as cervical intraepithelial neoplasia (CIN), to progress through stages CIN1, CIN2, and CIN3 before becoming invasive cervical cancer; further, 43% of CIN2 and 32% of CIN3 lesions regress and do not become cancerous [16]. A group of women with proper treatment experienced only 0.7% of the CIN3 lesions becoming invasive cancers (within 30 years), as compared to 31% without treatment [17]. Thus, the screening programs allow for early detection and treatment and provide motivation to achieve full participation in the programs. As there are cases where screening is not used and invasive cervical cancer may develop, it is of value to consider what risk factors impact the progression of the precancerous lesions to the stage of invasive cancer.

The National Health Interview Survey has demonstrated that, in regard to screening, socioeconomic status, income, and education are more relevant than race and ethnicity [26]. Due to the nature of the disease and the treatment, differences in mortality arise largely from late stage at presentation, a factor related to failure in screening. The racial, ethnic, and socioeconomic disparities in cervical cancer survival can be explained by late-stage presentation and less than effective treatment [27]. The difference in survival between Black and White women apparently does not originate in biological differences but rather in exposure to HPV and access to screening and treatment. In a stage-for-stage comparison, there are no differences in survival [28], and, with equal treatment, survival is independent of race [29], [30]. Further, race is not an independent prognostic factor for the response of cervical cancer patients to radiation therapy [31].

A late presentation of the disease, leading to excessive mortality, should be considered a failure of the screening process [15]. The higher rates of HPV infection and mortality among the lower socioeconomic classes result from a complex interaction of numerous causes, some of which are not fully understood. Efforts have been made to clarify the reasons for under-coverage of certain segments of the population and to determine solutions that will improve outcomes [32], [33]. The differences in access to screening and treatment between socioeconomic classes directly impact survival [34] and point to the need to address the disparity in insurance and access to service. These issues are magnified by some patients refusal of treatment and failure to follow the treatment plan [15] and by distrust or cultural issues related to screening and treatment [35].

Due to various factors, Black women are less likely to receive surgical treatment [27], [36], [37]. Knowledge of risk can also be an issue, as detailed in a subset of the population with high risk but without screening and without awareness of risk [38]. The lower socioeconomic classes also have higher incidences of comorbid conditions [27], [35], which not only increase the risk of progression of the disease but also lead to lower screening rates [39]. Differences in the prevalence of HPV DNA among different geographical regions and different segments of the population within a region have particular relevance [40]. Within this issue, there is interaction between our study of the variable of race and our longitudinal study among the different states. Nevertheless, further studies are necessary to determine gradients in rates of incidence, stage, and mortality over geographic regions and the relation of race, ethnicity, and socioeconomics in order to design better screening and treatment strategies [41].

This aspect of our statistical/mathematical model, treating the gradient of the racial difference in the mortality rate with respect to different geographical regions, allows for interpretations regarding the source of the regional variation in this disparity. One interpretation is that the underlying socioeconomic disparities corresponding to the racial differences operate at different levels in these different regions [40]. Also relevant is the observation that regional variation in the prevalence of HPV DNA is associated with these differences [40]. The SEER data indicates distinct regional differences in infection and mortality rates, with Delaware, Florida, Louisiana, Mississippi, Oklahoma, and West Virginia exhibiting poor performance for both of these measures [11].

The racial and ethnic disparities for cervical cancer are of concern for issues of equity and fairness, for issues of how to improve treatment, and for identifying regions where efforts to combat cervical cancer are more or less effective. These disparities and socioeconomic factors extend to the other gynecological cancers and to other types of cancers [42], [43], [44]. Equity and removal of disparities are a concern within the healthcare system [45], [46], [47]. The case of cervical cancer calls for attention to disparities since effective screening and treatment exist, and the associated suffering, costs, and mortality should be avoidable. There is an overlap with preventive medicine, the most efficient and desirable form of medicine. A goal will be to use these models to understand the source of the racial disparity for survival in cervical cancer. Since HPV infections and cervical cancer are preventable and treatable, success will be achieved by distributing the screening and treatments to all segments of the U.S. population.

Although there are numerous causes for disparities, including socioeconomics, the present results address the pace of decline in cervical cancer mortality across states and its relation to racial disparities. Similar, and even greater, disparities exist on the global scene. Doctors and health officials have made progress and continue to address these disparities within the U.S. population; still, the approach should be extended globally.

We note that screening programs instituted in the U.S. and other developed countries have been highly effective and have avoided much suffering and loss of life, including an estimated 6,000 lives per year in the U.K. [48]. Most of the burden of cervical cancer is in developing countries, where it ranks first or second among cancers for women; in developed countries, it is not in the top five [49], [50]. Of the 528,000 new cases each year, 70% to 80% occur in developing countries [15], [51]. Rates in India and in African countries can be ten to twenty times higher [52]. The ravages of cervical cancer in these countries can be attributed primarily to the lack of screening [15]. While an overall goal within medicine includes extending to the larger world increases in survival and life quality from medical advances in developed countries, this case should be of particular relevance due to significant differences in outcomes and relative ease of treatment. For instance, the suggestion to extend current cancer control knowledge to the lowest socioeconomic bracket [42] is relevant in regard to lives that can be saved in the developing world. Two vaccines, Gardasil and Cervarix, have been developed for use in the U.S., and, if accomplished, utilization of these vaccines worldwide is estimated to reduce cases of cervical cancer up to 70% [53].

Despite overall decline in mortality, Black women continue to have higher mortality rates from cervical cancer than White women. The disparity in mortality may be related to poverty, lack of education, and socioeconomic factors. A report by the Centers for Disease Control and Prevention [54] indicates that, from 2000 through 2008, the percentage of Black women ages 18 years or older who had a Pap test in the last three years was higher than that for Whites. This may be a reason for the faster decline in cervical cancer mortality rate for Black women. Early detection through screening is a factor in increasing the pace of decline in cervical cancer mortality; direct consequences are the reductions in mortality rates for both races and a reduction of racial disparities. We believe that individual states should promote cervical cancer screening through effective media, such as television, and thereby encourage women to be screened for cervical cancer. Such promotions can increase cervical cancer awareness and point out the risks of inaction. To have the most impact on encouraging women to schedule regular cervical cancer screenings, the promotions should be prepared jointly by public health and marketing professionals.

## Conclusions

The results of the present study show that disparities exist for cervical cancer. Although Black women have higher mortality rates, the pace of decline in their mortality is higher than that for White women. This increase in pace, if sustained, will reduce the racial disparities in cervical cancer. Public health officials should monitor progress toward elimination of these disparities.
